# Mediterranean Diet Modulation of Neuroinflammation-Related Genes in Elderly Adults at High Cardiovascular Risk

**DOI:** 10.3390/nu16183147

**Published:** 2024-09-18

**Authors:** Javier Hernando-Redondo, Mireia Malcampo, Karla Alejandra Pérez-Vega, Indira Paz-Graniel, Miguel Ángel Martínez-González, Dolores Corella, Ramón Estruch, Jordi Salas-Salvadó, Xavier Pintó, Fernando Arós, Inmaculada Bautista-Castaño, Dora Romaguera, José Lapetra, Emilio Ros, Raquel Cueto-Galán, Montserrat Fitó, Olga Castañer

**Affiliations:** 1 CIBER de Fisiopatología de la Obesidad y Nutrición, Instituto de Salud Carlos III, 28029 Madrid, Spain; jhernando1@researchmar.net (J.H.-R.); kperez@researchmar.net (K.A.P.-V.); indiradelsocorro.paz@urv.cat (I.P.-G.); mamartinez@unav.es (M.Á.M.-G.); jordi.salas@urv.cat (J.S.-S.); lfaborau@gmail.com (F.A.); eros@clinic.cat (E.R.); 2 Unit of Cardiovascular Risk and Nutrition, Hospital del Mar Medical Research Institute, 08024 Barcelona, Spainocastaner@researchmar.net (O.C.); 3 Ph.D. Program in Food Science and Nutrition, University of Barcelona, 08028 Barcelona, Spain; 4 Departament de Bioquímica i Biotecnologia, Alimentació, Nutrició, Desenvolupament i Salut Mental ANUT-DSM, Universitat Rovira i Virgili, 43201 Reus, Spain; 5 Department of Preventive Medicine and Public Health, Instituto de Investigación Sanitaria de Navarra (IdiSNA), Universidad de Navarra, 31009 Pamplona, Spain; 6 Departament of Preventive Medicine, University of Valencia, 46010 Valencia, Spain; 7 Departament of Internal Medicine, Institut d’Investigacions Biomèdiques August Pi i Sunyer (IDIBAPS), Hospital Clínic, University of Barcelona, 46010 Barcelona, Spain; 8 Lipids and Vascular Risk Unit, Internal Medicine, Institut d’Investigació Biomèdica de Bellvitge (IDIBELL), Hospital Universitario de Bellvitge, University of Barcelona, 08028 Barcelona, Spain; 9 Cardiology Department, Organización Sanitaria Integrada Araba (OSI ARABA), University Hospital of Araba, 01009 Gasteiz, Spain; 10 University of País Vasco/Euskal Herria Unibersitatea (UPV/EHU), 01006 Vitoria-Gasteiz, Spain; 11 Institute for Biomedical Research, University of Las Palmas de Gran Canaria, 35001 Las Palmas de Gran Canaria, Spain; inmaculada.bautista@ulpgc.es; 12 Research Group in Nutritional Epidemiology and Cardiovascular Pathophysiology, Instituto de Investigación Sanitaria Illes Balears (IdISBa), 07120 Palma de Mallorca, Spain; 13 Department of Family Medicine, Research Unity, Distrito Sanitario Atención Primaria Sevilla, 41013 Seville, Spain; 14 Institut d’Investigacions Biomèdiques August Pi i Sunyer (IDIBAPS), Hospital Clínic, 46010 Barcelona, Spain; 15 Preventive Medicine and Public Health Department, School of Medicine, University of Malaga, Spain, Biomedical Research Institute of Malaga (IBIMA), 29071 Malaga, Spain; rcueto@uma.es; 16 CIBER de Epidemiología y Salud Pública, Instituto de Salud Carlos III, 28029 Madrid, Spain

**Keywords:** Mediterranean diet, neuroinflammation, cardiovascular disease, nutrigenomics

## Abstract

Individuals with dementia and neurodegenerative diseases (NDDs) often suffer from cardiovascular diseases (CVDs). Neuroinflammation driven by conditions involved in CVDs is linked to disruptions in the central nervous system triggering immune reactions, perpetuating an “inflammatory-like” environment. The Mediterranean diet (MedDiet), known for its anti-inflammatory and antioxidant properties, has been proposed as a key factor to attenuate these risks. Blood nuclear cell samples were collected from 134 participants of the PREDIMED trial, which randomized participants to three diets: one supplemented with extra-virgin olive oil (MedDiet-EVOO), another with nuts (MedDiet-Nuts), and a low-fat control diet. These samples were analyzed at baseline and 12-month follow-up to assess the impact of these dietary interventions on gene expression markers. We first selected target genes by analyzing intersections between NDD and CVD associations. Significant gene expression changes from baseline to 12 months were observed in the participants allocated to the MedDiet-EVOO, particularly in CDKN2A, IFNG, NLRP3, PIK3CB, and TGFB2. Additionally, TGFB2 expression changed over time in the MedDiet-Nuts group. Comparative analyses showed significant differences in TGFB2 between MedDiet-EVOO and control, and in NAMPT between MedDiet-Nuts and control. Longitudinal models adjusted for different covariates also revealed significant effects for TGFB2 and NAMPT. In conclusion, our results suggest that one year of traditional MedDiet, especially MedDiet-EVOO, modulates gene expression associated with CVD risk and NDDs in older adults at high CV risk.

## 1. Introduction

A significant proportion of patients affected by dementia have common comorbidities such as cardiovascular diseases (CVDs), type 2 diabetes, hypertension, dyslipidemia or excess body weight. CVDs and neurodegenerative diseases (NDDs) share risk factors that predispose and accelerate both pathologies [1]. One critical non-modifiable pro-inflammatory factor that must be considered is inflammaging [2], a term referring to the chronic progressive stress caused by aging, which increases inflammatory status. Inflammaging added to cardio metabolic risk factors first triggers immune responses in peripheral organs, initiating a systemic inflammatory state that later affects the central nervous system (CNS) and eventually disrupts homeostasis in both areas [3,4]. Once inflammatory cells, molecules, or cytokines infiltrate the CNS, resident glial cells are activated, perpetuating neuroinflammation [3,5,6,7].

Neuroinflammation is characterized by a primary immune reaction to brain injury mediated by key pro-inflammatory cytokines, particularly interleukin (IL)-1b, IL-6, and tumor necrosis factor (TNFα). The neuroinflammatory process involves the activation and priming of glial cells, during which microglia exert macrophage-like functions such as vital surveillance, scavenging, antigen presentation, and cell repair [8]. The released cytokines increase blood–brain barrier (BBB) permeability [9], thereby increasing their own concentrations in the brain, which intensify microglia’s pro-inflammatory responses [10]. The type, degree, and duration of the stimulus determine the neuroinflammatory damage responsible for the destruction of brain tissues characteristic of NDDs [10,11,12]. NDDs are influenced by a wide range of factors such as age, sex, nutrition, socio-economic, and genetic determinants, in addition to lifestyle habits (physical activity, metal health, and wellbeing) that interplay in triggering or accelerating the disease [12,13,14].

It is known that there are components in Mediterranean dietary patterns that exert a protective effect, contributing to attenuate the neuroinflammatory state [12,15]. Resveratrol, tyrosol, anthocyanins, and isoflavones, found in common sources of the Mediterranean diet (such as olive oil, fruits, and legumes), have been associated with varying degrees of consistency in improving cognitive function. From a mechanistic point of view, they exert anti-inflammatory, anti-apoptotic, and neuroprotective effects by modulating various pathways involved in oxidative stress, inflammatory mediators, and promoting cell survival mechanisms [16]. Further, the gut–brain axis has recently been proved as a key entity influencing multiple states or diseases, being susceptible to modification by a variety of factors including diet, drug intake, or medical procedures [17].

Epidemiological studies have tried to bridge the gap between CVDs and Alzheimer’s disease (AD) through association studies, identifying common variants of both diseases [18,19,20]. Additionally, research on the transcriptional profile has inferred genetic information from blood cell analysis, reflecting primary alterations occurring in tissue. Cell-free RNA has been demonstrated to distinguish AD patients versus age-matched controls by comparing transcript levels of AD-related genes [21]. In this regard, advancements in this field have revealed correlations between disease severity and the characteristics of circulating transcriptomes [22].

Thus, systemic inflammation and neuroinflammation play pivotal roles in the progression of NDDs, wherein both established chronic inflammatory processes contribute to brain damage and cognitive impairment. MedDiets may benefit brain function by attenuating both neuroinflammation and systemic inflammation, measured through different gene biomarkers linked to these pathological processes.

The aim of this sub-study of the PREDIMED randomized trial [21] was to determine the effect of a long-term Mediterranean diet (MedDiet) intervention on the gene expression of transcriptomic biomarkers related to neuroinflammation and cardiovascular risk in an older population at high cardiovascular risk.

## 2. Materials and Methods

### 2.1. Study Design and Population Recruitment

The study population was a random subsample of volunteers (*n* = 134, 67 men and 67 women) recruited in different sites of the large-scale multicenter randomized, controlled trial PREDIMED (flow chart in Figure S1 Supplementary Material). PREDIMED assessed the effect of MedDiets on the primary prevention of CVD [23]. In this trial, two traditional MedDiets were tested for long-term effects on CVD risk, one enriched with extra-virgin olive oil (MedDiet-EVOO) and another enriched with raw nuts (MedDiet-Nuts) vs. a control diet based on advice to reduce the fat content of the diet.

Eligible participants were women and men, aged 60–80 years and 55–80 years, respectively, who met at least one of the following criteria: (1) type 2 diabetes or (2) ≥3 major cardiovascular risk factors: current smoking (>1 cig/day during the last month); hypertension (systolic BP ≥ 140 mmHg or diastolic BP ≥ 90 mmHg or antihypertensive medication); LDL cholesterol ≥ 160 mg/dL or lipid-lowering therapy; HDL cholesterol ≤ 40 mg/dL in men or ≤50 mg/dL in women; body mass index ≥ 25 kg/m^2^; and family history of early-onset coronary heart disease [23]. Exclusion criteria were a prior history of CVD, severe chronic illnesses, substance abuse, allergies or intolerance to olive oil or nuts, a low predicted likelihood of changing dietary habits based on the stages of change model [24], or any condition that might hinder study participation.

### 2.2. Blood Chemistry Analysis

Sample collection was performed after an overnight fast at baseline and after a 12-month follow-up. Collected samples were centrifuged immediately after extraction, both for 15 min at 1.700× *g* room temperature. The following analytes were quantified in serum with an ABX Pentra-400 auto-analyzer (Horiba-ABX, Montpellier, France): glucose (mg/dL), triglycerides (mg/dL), HDL-cholesterol (mg/dL), and total cholesterol (mg/dL). LDL-cholesterol was calculated according to the Friedewald formula when triglycerides were <300 mg/dL.

### 2.3. Cardiovascular and Lifestyle Factors

Dyslipidemia was defined as meeting any of the following criteria: HDL-cholesterol < 40 mg/dL or 50 mg/dL (for men and women respectively), LDL-cholesterol > 200 mg/dL, triglycerides > 150 mg/dL or taking any lipid-lowering drugs. Adherence to the MedDiet was assessed by a validated 14-item questionnaire [25]. Physical activity was recorded via the Minnesota leisure time physical activity questionnaire [26,27].

### 2.4. RNA Extraction, Reverse Transcription, and Gene Expression Analysis

Nuclear cells were extracted from peripheral blood by using tubes for purification of intracellular RNA from human whole blood (range of white blood cells 4.8 × 10^6^–1.1 × 10^7^ leukocytes/mL) for in vitro diagnostics applications (PAXgene Blood RNA Tube, BRT, Hombrechtikon, Switzerland). The RNA concentration (A260) and purity were calculated spectrophotometrically (NanoDrop ND-1000; NanoDrop Technologies, v3.5, Wilmington, NC, USA). RNA integrity was assessed by using microcapillary gel electrophoresis (Bioanalyzer, NanoChip; Agilent Technologies, version 2.6, Waldbronn, Germany) and the RNA integrity number value (RIN) was calculated with Agilent 2100 Expert Software (Agilent Technologies).

Low-input samples (50–200 ng/µL) underwent preamplification because recommendations pointed target levels should be above 200 ng/µL. Amplification was performed using TaqMan^®^ PreAmp Master Mix (Applied Biosystems, Vilnius, Lithuania). Reverse transcription to cDNA was carried out with a High-Capacity cDNA Reverse Transcription Kit with RNase Inhibitor (Life Technologies, Vilnius, Lithuania). Microarray RT-PCR step was performed using a QuantStudio™ 12K Flex Real-Time PCR System (Life Technologies) and TaqMan^®^ OpenArray™ Real-Time PCR Master Mix (Aplied Biosystems, Vilnius, Lithuania). Finally, results were analyzed with QuantStudio™ 12K Flex Software version 1.3.

### 2.5. Selection of Gene Targets

**Figure 1:**
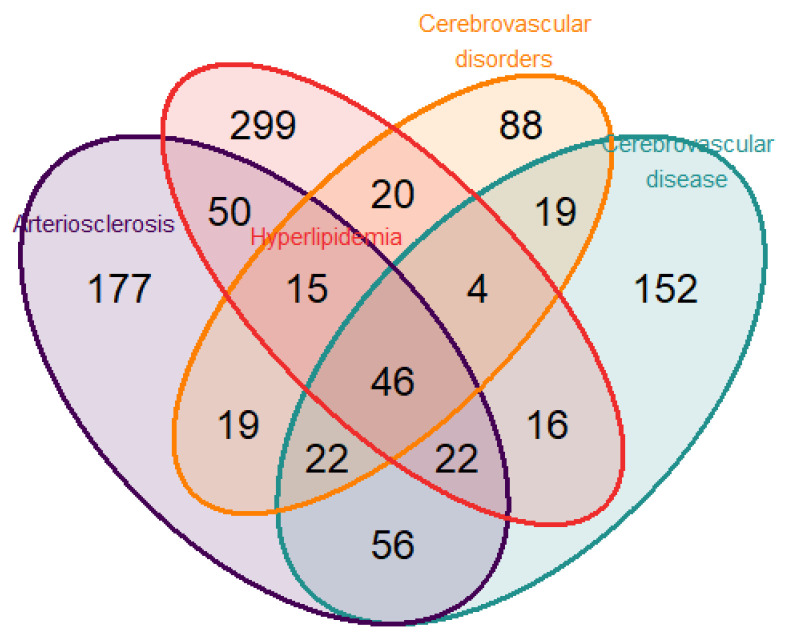
Venn diagram displaying the number of genes found from the overlap of the 4 selected pathologies (atherosclerosis, hyperlipidemia, cerebrovascular disorders, and cerebrovascular disease) employing the public databases DisGeNET and Disease Ontology.

To identify genes related to both neurodegenerative disease and CVD, we performed a disease enrichment analysis to search human gene–disease interactions. The search was executed including the following terms: “atherosclerosis”, “cognitive”, “cholesterol”, “lipid”, “metabolism”, “cerebrovascular”, “dementia”, and “arteriosclerosis”. From the intersection of the selected diseases, “Arteriosclerosis” and “Cerebrovascular disease” in DisGeNET (Database of Gene-Disease Associations) v6 and v7 [28] and “Hyperlipidemia” and “Cerebrovascular disorders” in the Human Disease Ontology [29] (Figure 1), a total of 46 genes were identified. For the present study, the nine genes selected from the intersection according to their biological functions were (a) IFNG (Interferon Gamma); IL10 (Interleukin 10); NFE2L2 (Nuclear Factor, Erythroid 2 Like 2); NLRP3 (NLR Family Pyrin Domain Containing 3); TGFB2 (Transforming Growth Factor Beta 2); (b) Regulators of the senescence and cell cycle: CDKN2A (Cyclin Dependent Kinase Inhibitor 2A); (c) Metabolic and Cell Signaling Regulators: PIK3CB (Phosphatidylinositol-4,5-Bisphosphate 3-Kinase Catalytic Subunit Beta); and NAMPT (Nicotinamide Phosphoribosyltransferase).

### 2.6. Reference Genes and Relative Quantification

We employed the relative quantification approach to present the analysis of gene expression data. We tested 3 potential candidates as control genes (also known as reference or housekeeping genes). The reference genes were chosen using the geNorm algorithm, which conducted a preliminary analysis to distinguish among 21 candidates. The algorithm tested gene expression variation across different samples, resulting in genes with lower M values, or those that were more stably expressed and, therefore, better candidates: we selected GAPDH as a reference. To study differences between baseline value and 12-month follow-up we compared ∆Ct.

The efficiency of the target and reference genes was 100% ± 10% [30,31,32], we applied the 2^−ΔΔCt^ method to quantify changes in gene expression. Therefore, the data are presented as a fold change value normalized to the reference gene and relative to the baseline value. Each pair of patient samples was allocated in the same plate to remove potential run-to-run variation [33,34,35,36].

### 2.7. Statistical Analyses

The sample size of 36 participants allowed at least 80% power to detect statistically significant differences in PIK3CB gene expression among the 3 groups, within the PREDIMED study of 0.4 units of the relative quantification log2FC, assuming a 2-sided type error of 0.05. A common standard deviation (SD) of 0.6 was estimated.

The assessment of the normality distribution was conducted using normality probability plots and boxplots. Descriptive statistics (mean and standard values) and comparison were calculated at baseline, post-intervention, and 12-month change to display nutritional parameters, energy intake, and key food components. The MedDiets were compared with the control diet using independent Student *T*-tests. We compared individuals’ changes along the intervention. To assess inter-group differences, independent Student *T*-tests were calculated employing ΔCt values. To account for the effect of relevant covariates, we performed two models: the first one was adjusted for age, sex, time, and education level; and the second one was additionally adjusted for diabetes, BMI, physical activity, smoking status, hypertension, and dyslipidemia. Diabetes and hypertension were treated as binary qualitative variables, categorized as either ‘Yes’ or ‘No’. Smoking status was stratified as a qualitative variable with three categories: current smoker, former smoker, and never smoker. Dyslipidemia was previously defined as a composite variable based on HDL-cholesterol, LDL-cholesterol, triglyceride levels, or lipid-lowering drugs. Educational level was a qualitative variable comprising 3 categories: higher education or equivalent, secondary education, or primary education. Physical activity was measured as a continuous variable in MET-minutes per week (METmin/week). BMI was also treated as a continuous quantitative variable, expressed in kg/m^2^. The interaction term time:group of intervention to explain inter-group variability across the trial. Inter-individual variability was assessed through random intercept. The linear mixed-effect model was estimated using restricted maximum likelihood. We used the lme function from the package nlme, using R software version 4.3.2 [37].

## 3. Results

The mean age and standard deviation of the study population was 66.1 (±6.34) years. Table 1 displays cardiovascular risk factors stratified per group of intervention. Among the subsample, 53% of participants had diabetes, 57% had dyslipidemia, 79% had hypertension, and 15% were smokers. Baseline characteristics of the groups were similar to those of the entire PREDIMED cohort (Supplementary Table S1). We discarded a total of 17 samples due to technical issues during the experimental phase of RT-qPCR.

Energy intake, nutritional parameters, and key food components are summarized in Supplementary Table S2. No between-group differences in 12-month change in energy intake were found. Between-group 12-month differences in changes were observed for total fat intake, mainly due to monounsaturated fatty acids (MUFA), between MedDiet-EVOO and MedDiet-Nuts compared to the control diet. We also observed differences in polyunsaturated fatty acids (PUFA) changes between the MedDiet-Nuts and control diet groups. The overall consumption of virgin olive oil and nuts were respectively increased in the MedDiet-EVOO and MedDiet-Nuts interventions compared to the control diet.

### Gene Expression

The 2^−ΔΔCt^ values correspondent to gene expression quantification (upregulation or downregulation) are depicted through divergent bars in a visual chart (Figure 1). In the MedDiet-EVOO intervention group, we observed significant 12-month changes in the following gene expression values: CDKN2A, IFNG, NLRP3, PIK3CB, and TGFB2. In this line, we observed temporal change in TGB2 in MedDiet-Nuts. Significant between-group differences in TGFB2 expression were found between the MedDiet-EVOO and control diet. Additionally, significant differences in NAMPT expression were observed in between-group comparisons of MedDiet-Nuts and the control diet group. Model 1 (adjusted for sex, age, and education level) resulted in statistically significant differences for the interaction term time-group of intervention in TGFB2 between the MedDiet-EVOO and control diet. The comparison between the MedDiet-Nuts and control diet disclosed significant results in NAMPT. Meanwhile, model 2 only showed significant differences by statistical criterion in NAMPT in MedDiet-Nuts. The results of the statistical analyses, including within-group, between-group comparisons, and model outputs, are presented in Supplementary Table S3.

## 4. Discussion

**Figure 2:**
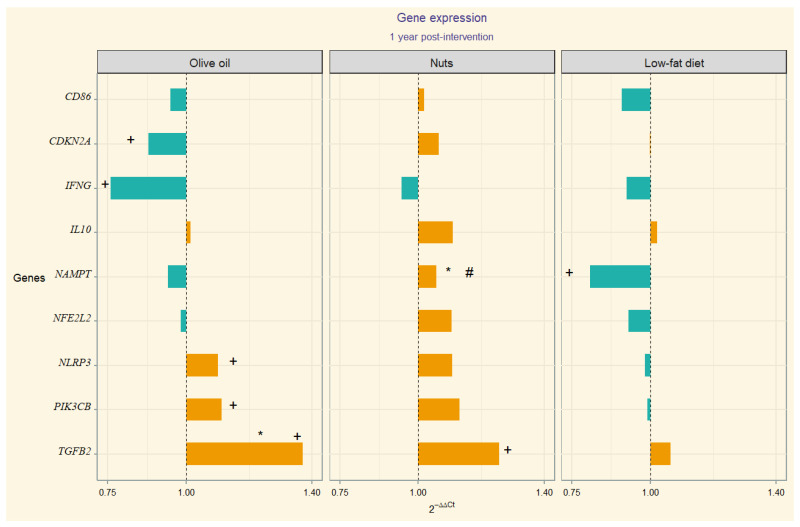
Divergent bar showing 2^−∆∆Ct^ per group (Orange bars represent upregulation of gene expression, while blue bars indicate downregulation). Statistically significant (*p*-value < 0.05): + baseline to post-intervention change; * individual comparison between MedDiet-EVOO and MedDiet-Nuts versus control; # time:group interaction (*p*-value) from mixed-effects of the fully adjusted model compared to control. Numerical *p*-values are presented in Supplementary Table S3.

In the search of the molecular mechanisms by which a cardioprotective diet like the Mediterranean one benefits brain health, we analyzed neuroinflammation- and systemic inflammation-related genes’ behavior after a Mediterranean diet intervention. Specifically, the MedDiet-EVOO modulated the expression of the CDKN2A, IFNG, NLRP3, PIK3CB, and TGFB2 genes in the peripheral blood mononuclear cells of older adults at high cardiovascular risk, whereas the MedDiet-Nuts resulted in a different expression of the NAMPT gene compared to the control diet (Figure 2).

There is evidence that AD patients display high plasma levels of numerous pro-inflammatory markers [38,39], including IFN-γ protein levels [40]. In this line, it is known that TGFβ interacts with IFN-γ, indicating a crosstalk between these pleiotropic cytokines, functioning as reciprocal regulation [41]. TGF-β’s signaling is involved in multiple neurological pathways, regulating synaptic growth, neurotrophic functions, and cell survival, although their role is not completely understood, especially in AD [42,43,44]. Research on the role of TGF-β1 in dementia [41,42,44] has shown reduced plasma concentrations in patients with AD [45].

Despite that Aβ42 levels rise at the onset of AD, as the disease progresses, they typically decrease [46,47]. In the present study, we observed an increased expression of TGF-β2 (Figure 2), in particular after the MedDiet interventions, suggesting a possible protective role in this older population. This is aligned with some limited evidence that points at a neurotrophic effect [48], although TGF-β2 functions remain unclear. However, prior research has shown that in AD patients, neurons bearing neurofibrillary tangles exhibit increased expression of TGF-β2 compared to cognitive age-matched controls [49,50,51]. There is limited evidence, based on small studies, that TGF-β2 may play a deleterious role in AD, justified by the higher protein levels found in the brains of AD patients [50,52], but on the other hand, there seems to be a reduced presence of receptors in the neurons of AD patients [51,53]. Our results may be in favor of the effect of the Mediterranean diet through the TGFβ2 anti-apoptotic effect described in other cell types [54].

The inflammasome is a multiprotein complex of the innate immune system that detects danger signals such as tissue damage, cellular stress, or infection. Cytokines become active by inflammasome mediation, triggering local and systemic inflammatory responses essential for immune defense but also involved in chronic inflammatory diseases when deregulated [55]. The NLRP3 inflammasome plays a pivotal role in the onset and progression of Aβ in mice, and may participate in protein tau pathology as well [56]. Prior research conducted to assess the effect of MedDiet adherence counteracting neuroinflammation and age-related diseases revealed that a 3-month intervention with a MedDiet supplemented with different olive oils was associated with downregulation of IFNG transcriptomic levels [57,58], among other inflammatory and proatherogenic biomarkers [59]. In a long-term (3 years) transcriptomic PREDIMED sub-study, a downregulation of both IFN-γ and NLRP3 was reported [60].

Hormesis is based on exposure to a substance exerting a biphasic response depending on the dose [61,62]. This may explain possible differences regarding the gene expression modulation by diet, specifically the upregulation pattern observed in MedDiet-EVOO (Figure 2). The inter-species hormesis theory postulates that the stress-induced synthesis of plant polyphenols, among other phytochemicals, can produce an environmental chemical signature that leads to stress resistance in other species [63,64,65]. In this regard, an hormetic dose-response behavior regarding NLRP3 has been described considering polyphenol intake [66,67,68] that aligns with this hypothesis, suggesting that hormesis may explain these findings.

NAMPT is the gene encoding visfatin, a dual-form ubiquitously expressed, whose functionality encompasses multiple processes related to insulin sensitivity, NDDs, lipid metabolism, atherosclerosis, and pro-inflammatory effects [69,70,71], including NLRP3 inflammasome activation [72,73]. It has also been described that NAMPT promotes IFN-γ secretion in CD4+ T lymphocytes, but paradoxically the IFNG transcript was upregulated when NAMPT was inhibited, suggesting a posttranslational level regulation [74]. An influence of diet on NAMPT expression has been described [75,76]. With regard to visfatin circulating levels, a decrease after a hypocaloric diet with significant weight loss has been described [77]. It has also been reported that the fat type can influence the visfatin response [78,79]. In this regard, no significant weight change was apparent with the MedDiet interventions in the full PREDIMED cohort [80]. Nevertheless, we have observed a significant downregulation of NAMPT in the control diet group compared to the MedDiet supplemented with nuts (Figure 2).

In the present study, the MedDiet-EVOO was the unique group in which an upregulation of PIK3CB and downregulation of CDKN2A versus baseline was observed (Figure 2). In one RNA-seq data study, the mean expression of PIK3CB in AD patients was lower than that of controls, suggesting a role in AD pathogenesis through apoptosis [81]. Similarly, altered blood expression of CDKN2A, measured by qRT-PCR, was reported in preclinical AD patients compared to controls [82]. The CDKN2A protein has previously been described to be upregulated in the brains of patients with AD [83]. These findings suggest that whole blood could be an emerging valuable tissue for unraveling the pathophysiology of AD and differentiating it from normal aging [84].

Several meta-analyses conducted in older adults (60–80 y) have concluded that greater MedDiet adherence usually correlates with an overall better cognitive performance [85,86,87]. Nevertheless, the evidence for causality is weak. The Three-City cohort is a French longitudinal (4-year follow-up) study designed to assess the risk of dementia, with almost 10,000 older participants (mean age, 74 years) [88,89], which did not demonstrate an association between adherence to the MedDiet and the risk of developing dementia [90].

The disbalance between oxidative stress and antioxidant systems has been suggested to play a role in the pathophysiology of NDDs. Further evidence is required to demonstrate if MedDiet can exert a protective effect on dementia or delay its onset [91,92] and explore the underlying molecular mechanisms of these benefits.

### Strengths and Limitations

The first limitation is the relatively small sample size, which may result in limited statistical power. More repeated measurements are required to study the dynamic behavior of molecular mechanisms, and therefore improve the strength of the conclusions. As a consequence, the study may not have sufficient sensitivity to identify relevant associations between the variables of interest. The second limitation is that cognitive impairment and risk of dementia were not primary endpoints in the PREDIMED study. No cognitive tests were performed, even though alternative subcohorts within the PREDIMED study have evaluated such outcomes. The third limitation is the biological specimen employed in the transcriptomic study, taking into account that gene expression varies depending on the tissue analyzed. In this regard, PBMCs from blood are useful for studying cardiovascular biomarkers such as inflammation and peripheral cholesterol efflux. However, blood is not a good indicator for selective gene expression in other tissues. Although neurons and cerebral microvasculature cells are closely related to neurodegenerative risk, collecting these cells in population-based research is impractical. Finally, our cohort comprised participants at high cardiovascular risk. Thus, results may not be generalized to the average elderly population.

### 5. Conclusions

Our results suggested that the expression of inflammatory pathways-related genes linked to both CVD and NDDs is moderately modulated by a traditional MedDiet, especially when supplemented with extra-virgin olive oil, in circulating peripheral blood nuclear cells of older adults at high cardiovascular risk. In particular, the MedDiet-EVOO modulated the expression of CDKN2A, IFNG, NLRP3, PIK3CB, and TGFB2 genes, whereas the MedDiet-Nuts differently expressed NAMPT compared to the control diet. The underlying molecular mechanisms could explain the brain benefits of a cardioprotective diet, such as the Mediterranean diet, although more evidence on large-sized and long-term lifestyle interventions is needed. The research on biomarkers may discover specific molecules able to assess neuroinflammatory process in an accessible way. The combination with imaging techniques may enhance the detection and monitoring of these processes, offering a more comprehensive approach to diagnosis and treatment strategies for neuroinflammatory conditions.

## Figures and Tables

**Table 1:** General characteristics of the study population. Values are expressed as a percentage (for categorical variables) and mean (and standard deviation) for quantitative continuous variables. MedDiet-EVOO, Mediterranean diet supplemented extra virgin olive oil; MedDiet-Nuts, Mediterranean diet supplemented with nuts.

	All Participants (134)	MedDiet-EVOO	MedDiet-Nuts	Control
**Age (years. mean ± SD)**	65.82 ± 6.29	65.61 ± 5.49	66.10 ± 6.93	64.73 ± 6.50
**Sex (% women)**	67 (50%)	29 (59.2%)	13 (33.3%)	25 (54.3%)
**Hypertension**	**All participants**	**MedDiet-EVOO**	**MedDiet-Nuts**	**Control**
No	28 (20.9%)	10 (20.48%)	10 (25.64%)	8 (17.39%)
Yes	106 (79.1%)	39 (79.59%)	29 (74.35%)	38 (82.6%)
**Diabetes**	**All participants**	**MedDiet-EVOO**	**MedDiet-Nuts**	**Control**
No	63 (47.01%)	20 (40.81%)	18 (46.15%)	25 (54.34%)
Yes	71 (52.99%)	29 (59.18%)	21 (53.84%)	21 (45.65%)
Dyslipidemia *	**All participants**	**MedDiet-EVOO**	**MedDiet-Nuts**	**Control**
No	56 (42.75%)	20 (40.82%)	18 (47.37%)	18 (40.91%)
Yes	75 (57.25%)	29 (59.18%)	20 (52.63%)	26 (59.09%)
**Tobacco use**	**All participants**	**MedDiet-EVOO**	**MedDiet-Nuts**	**Control**
Current smoker	20 (14.93%)	9 (18.37%%)	6 (15.38%)	5 (10.87%)
Former smoker	38 (28.36%)	9 (18.37%%)	14 (35.90%)	15 (32.61%)
Never smoker	76 (56.72%)	31 (63.27%)	19 (48.72%)	26 (56.52%)
**Adherence to diet** **(14-point** **item score)**	8.72 ± 1.91	8.57 ± 2.02	8.62 ± 2.01	8.98 ± 1.72
**Physical activity** **(MET∙min/week)**	1906 ± 1670	1855 ± 1373	2232 ± 1995	1684 ± 1651

* Dyslipidemia is defined: HDL-cholesterol < 40 mg/dL or 50 mg/dL (for men and women respectively), LDL-cholesterol > 200 mg/dL, triglycerides > 150 mg/dL or taking any lipid-lowering drugs.

## Data Availability

The dataset analyzed during the current study cannot be made publicly available due to national data regulations and ethical considerations, including the absence of explicit written consent from study participants to make their deidentified data available upon study completion. However, data described in the manuscript will be shared with bona fide investigators for collaboration upon request. Requests for collaboration can be made by sending a letter to the PREDIMED Steering Committee (predimed-steering-committee@googlegroups.com).

## Supplementary Materials

The following supporting information can be downloaded at: https://www.mdpi.com/article/10.3390/nu16183147/s1, Figure S1: Flowchart of participant enrollment, randomization, and analysis in the PREDIMED; Table S1: Baseline characteristics of the subsample participants of PREDIMED (N = 134) and comparison with the correspondent baseline characteristics of the complete PREDIMED Study (N = 7237). Values are expressed as a percentage (for categorical variables), mean (standard deviation). Chi-Square test was performed for categorical variables and Student-T test for quantitative continuous variables. Table S2: Mean Consumption and Dietary Changes at Baseline, 12 Months, and Follow-up Comparison. Table S3: Student-T Test and Mixed-Effects Model Comparison of Gene Expression Changes Over 12 Months.
