# Supplementary material for: Mediterranean Diet Modulation of Neuroinflammation-Related Genes in Elderly Adults at High Cardiovascular Risk
Source: Nutrients. 2024 Sep 18;16(18):3147. doi: 10.3390/nu16183147 (PMC11434799; doi:10.3390/nu16183147)
Supplement: Supplementary file 1 [file nutrients-16-03147-s001.zip › nutrients-3217536-supplementary.pdf]

Supplementary material

Supplementary Figure S1. Flowchart of participant enrollment, randomization, and analysis in the PREDIMED

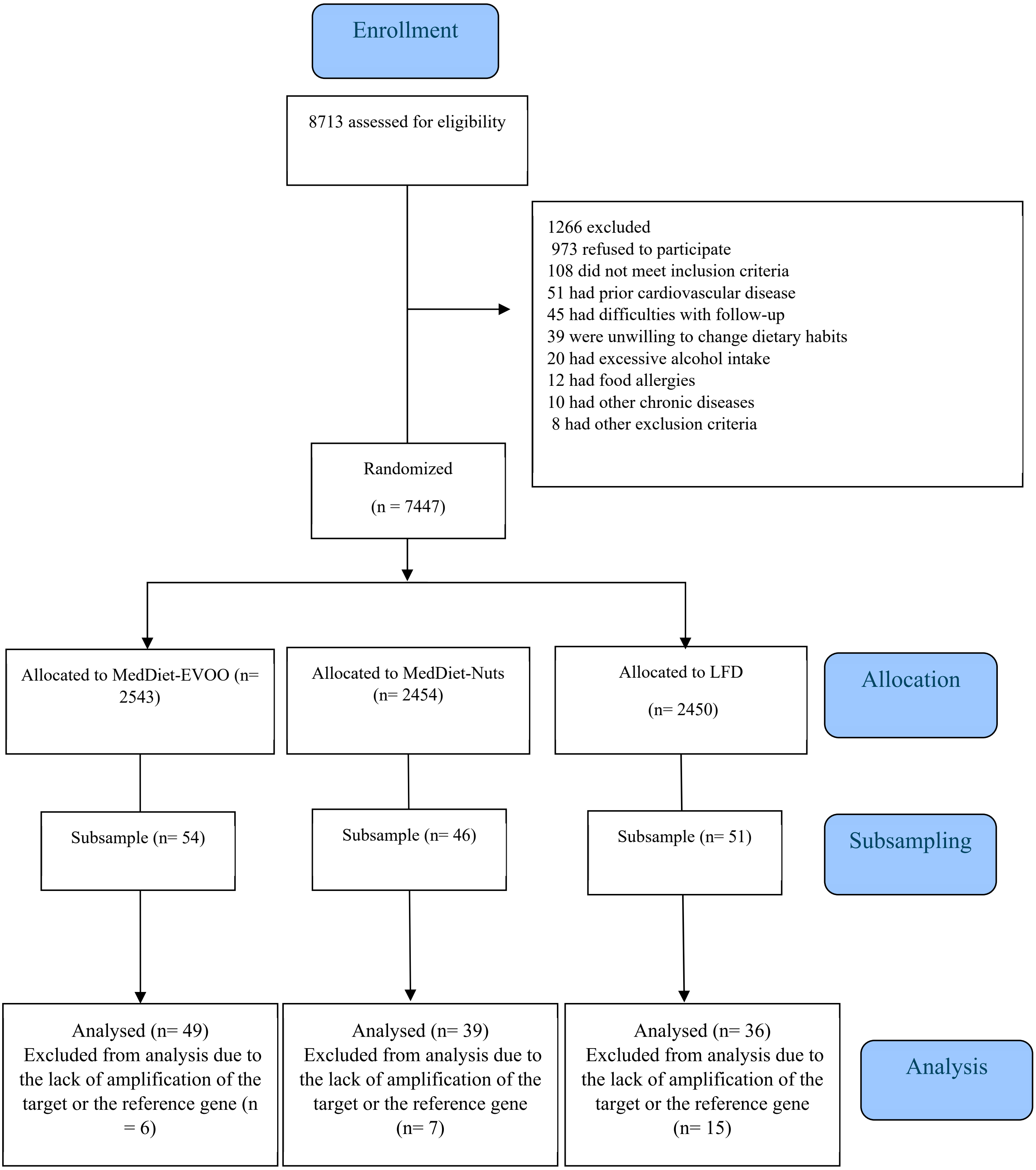

Supplementary Table S1. Baseline characteristics of the subsample participants of PREDIMED (N = 134) and comparison with the correspondent baseline characteristics of the complete PREDIMED Study (N=7237). Values are expressed as a percentage (for categorical variables), mean (standard deviation). Chi-Square test was performed for categorical variables and Student-T test for quantitative continuous variables.

	Sample (N = 134)	PREDIMED (N = 7313)	Confidence intervals
**Sex (%women)**	67 (50.0%)	4215 (57.6%)	(-0.001-0.012)
**Age**	65.8 (6.29)	67.0 (6.20)	(-2.291-(-0.117))
**Weight (kg)**	80.2 (12.6)	76.7 (11.9)	(1.280-5.628)
**BMI (kg/m^2)**	30.2 (3.67)	30.0 (3.85)	(-0.400-0.867)
**Waist circumference (cm)**	102 (9.48)	100 (10.3)	(0.125-3.424)
**Hypertension (% hypertensive)**	106 (79.1%)	6057 (82.8%)	(-0.004-0.013)
**Diabetes (% diabetic)**	71 (53.0%)	3561 (48.7%)	(-0.009-0.003)
**Dyslipidemia (%dyslipidemic)**	75 (57.3%)	3316 (48.0%)	(-0.013-0.000)
**Adherence to MedDiet (14-point item score)**	8.72 (1.91)	5.67 (4.40)	(2.713-3.397)
**Physical activity (MET·min/week)**	1906 (1670)	1608 (1675)	(10.777-586.495)
**Smoking status**			(0.095-0.223)
**Former smoker**	20 (14.92%)	1027 (14.04%)	
**Current smoker**	38 (28.35%)	1799 (24.6%)	
**Never smoker**	76 (56.71%)	4487 (61.35%)	

SD, standard deviation; MedDiet, Mediterranean diet; EVOO, extra-virgin olive oil; Dyslipidemia is defined by meeting any of the following criteria: HDL-c < 40 mg/dL or 50 mg/dL (for men and women respectively), LDL-c > 200 mg/dL, triglycerides > 150 mg/dL. * Three individuals were discarded for missing values

Supplementary Table S2. Mean and standard deviation in the consumption of key food items and dietary parameters of PREDIMED at baseline, 12 months and 12-month change. Mean of differences and confidence intervals from Student-T test comparison between groups at baseline, 12 months and 12-month change are presented. Mean of differences and confidence intervals from Student-T test comparison between groups at baseline and 12-month follow-up are presented.

	**MedDiet-EVOO**			**MedDiet-Nuts**			**Control**			**Baseline comparison to control**		**12 months comparison to control**		**12-month change comparison to control**	
	**Baseline**	**12 months**	**12-month change**	**Baseline**	**12 months**	**12-month change**	**Baseline**	**12 months**	**12-month change**	**MedDiet-EVOO**	**MedDiet-Nuts**	**MedDiet-EVOO**	**MedDiet-Nuts**	**MedDiet-EVOO**	**MedDiet-Nuts**
Energy intake (kcal/day)	2208.62 (524.79)	2152.81 (412.71)	-55.81 (570.03)	2211.03 (525.85)	2190.95 (476.61)	-20.08 (596.64)	2258.26 (501.67)	2078.13 (586.84)	-180.13 (560.66)	-74.62 (-258.78 - 159.50)	-82.97 (-270.30 - 175.83)	85.5 (-133.67 - 283.04)	86.3(- 116.66 - 342.30)	160.12 (-106.12 - 354.75)	169.27 (-91.42 - 411.53)
Carbohydrates (g/day)	235.64 (62.64)	219.39 (59.46)	-16.25 (77.39)	241.93 (74.84)	219.12 (68.17)	-22.81 (90.36)	232.95 (70.87)	222.66 (88.03)	-10.29 (79.13)	-3.83 (-24.64 - 30.03)	5.39 (-22.66 - 40.63)	-5.26 (-34.14 - 27.62)	-4.44 (-37.28 - 30.20)	-1.43 (-37.88 - 25.97)	-9.83 (-49.54 - 24.50)
Protein (g/day)	94.02 (26.20)	92.44 (18.17)	-1.58 (24.06)	89.73 (17.24)	87.97 (17.01)	-1.76 (19.35)	94.42 (17.72)	87.24 (17.89)	-7.18 (21.15)	-1.2 (-9.48 - 8.68)	-5.43 (-12.26 - 2.87)	5.7 (-2.15 - 12.55)	0.06 (-6.81 - 8.27)	6.9 (-3.62 - 14.82)	5.49 (-3.32 - 14.17)
Total fat (g/day)	93.47 (27.83)	95.34 (19.04)	1.87 (27.73)	91.41 (25.82)	100.51 (23.24)	9.10 (26.52)	98.63 (27.77)	85.52 (27.46)	-13.12 (26.41)	-4.93 (-16.50 - 6.17)	-8.72 (-18.80 - 4.35)	**11.39 (0.12 - 19.53)**	**13.35 (4.06 - 25.93)**	**16.32 (3.96 - 26.02)**	**22.07 (10.75 - 33.68)**
Saturated fatty acids (g/day)	23.64 (9.16)	21.75 (6.15)	-1.89 (7.31)	23.56 (7.79)	21.78 (7.29)	-1.78 (7.35)	25.63 (9.96)	20.43 (7.25)	-5.19 (9.54)	-1.83 (-5.89 - 1.92)	-2.23 (-5.90 - 1.77)	1.66 (-1.44 - 4.06)	0.8 (-1.80 - 4.50)	3.49 (-0.18 - 6.78)	3.03 (-0.23 - 7.06)
Monounsaturated fatty acids (g/day)	46.50 (14.14)	51.09 (10.25)	4.59 (14.04)	45.05 (12.77)	51.01 (11.97)	5.96 (13.57)	49.92 (15.02)	43.60 (15.42)	-6.32 (15.01)	-3.48(- 9.37 - 2.54)	-5.82 (-10.86 - 1.13)	**8.37 (2.10 - 12.87)**	**6.8 (1.49 - 13.32)**	**11.85 (4.97 - 16.84)**	**12.62 (6.10 - 18.44)**
Polyunsaturated fatty acids (g/day)	15.06 (6.23)	14.10 (4.00)	-0.95 (6.53)	14.42 (5.77)	18.26 (5.41)	3.84 (6.82)	14.43 (4.43)	13.39 (6.12)	-1.03 (5.90)	0.81 (-1.56 - 2.82)	-0.43 (-2.26 - 2.25)	0.96 (-1.42 - 2.84)	**4.53 (2.38 - 7.36)**	0.15 (-2.45 - 2.61)	**4.96 (2.10 - 7.66)**
Meat and meat products (g/day)	133.79 (55.41)	131.78 (51.14)	-2.01 (52.96)	119.91 (42.71)	108.36 (49.94)	-11.55 (43.42)	141.02 (51.90)	123.57 (47.50)	-17.45 (58.71)	-7.91 (-29.09 - 14.63)	-19.49 (-41.53 - (-0.70))	9.28 (-11.88 - 28.31)	-16.28 (-36.37 - 5.95)	17.18 (-7.40 - 38.28)	3.21 (-16.19 - 27.99)
Fish (g/day)	107.68 (54.42)	115.20 (47.25)	7.52 (53.25)	94.00 (41.86)	104.78 (41.22)	10.77 (51.73)	109.67 (41.01)	116.32 (40.21)	6.64 (38.75)	-1.38 (-21.57 - 17.57)	-16.83 (-33.63 - 2.29)	1.19 (-18.96 - 16.72)	-10.38 (-29.20 - 6.11)	2.56 (-18.03 - 19.78)	6.46 (-15.94 - 24.20)
Vegetables (g/day)	385.05 (168.52)	373.10 (165.82)	-11.95 (157.29)	329.74 (151.87)	315.91 (123.08)	-13.83 (146.35)	372.41 (127.72)	374.83 (150.55)	2.42 (152.86)	14.99 (-48.09 - 73.37)	-58.4 (-103.94 - 18.61)	-2.87 (-66.20 - 62.73)	-64.55 (-117.96 - 0.13)	-17.86 (-77.57 - 48.83)	-6.14 (-80.93 - 48.43)
Cereals without potato (g/day)	139.23 (79.12)	129.76 (81.64)	-9.47 (107.09)	141.27 (83.67)	138.12 (94.28)	-3.14 (109.34)	124.97 (57.18)	139.13 (91.98)	14.16 (97.45)	8.47 (-13.77 - 42.28)	13.06 (-15.32 - 47.90)	-8.72 (-44.91 - 26.16)	0.14 (-41.39 - 39.38)	-17.19 (-65.31 - 18.05)	-12.92 (-62.40 - 27.80)
Dairy products (g/day)	369.50 (250.63)	361.32 (233.26)	-8.18 (179.79)	395.69 (228.52)	324.56 (212.02)	-71.13 (199.05)	383.07 (237.47)	280.97 (154.20)	-102.10 (163.04)	-20.11 (-113.01 - 85.88)	6.5 (-88.13 - 113.38)	**81.23 (0.13 - 160.58)**	37.15 (-37.94 - 125.13)	**101.34 (24.06 - 163.78)**	30.65 (-48.59 - 110.53)
Nuts (g/day)	8.63 (10.82)	6.85 (8.26)	-1.78 (11.74)	10.23 (11.45)	27.42 (9.98)	17.19 (15.80)	7.74 (9.67)	5.71 (9.62)	-2.02 (12.20)	1.36 (-3.28 - 5.07)	2.23 (-2.14 - 7.12)	0.96 (-2.53 - 4.80)	**21.07 (17.45 - 25.96)**	-0.4 (-4.64 - 5.13)	**18.84 (13.02 - 25.40)**
Fruit (g/day)	390.19 (163.96)	429.95 (233.09)	39.76 (242.56)	374.75 (174.70)	407.48 (190.65)	32.73 (182.42)	464.25 (228.36)	435.95 (296.14)	-28.30 (282.25)	-88.95 (-155.66 - 7.55)	**-90.41 (-176.58 - (-2.42))**	-35.97 (-115.17 - 103.17)	-33.09 (-134.54 - 77.61)	52.98 (-39.54 - 175.66)	57.32 (-40.19 - 162.26)
Legumes (g/day)	24.54 (13.75)	26.13 (12.89)	1.60 (18.07)	22.99 (11.51)	24.94 (10.47)	1.95 (14.03)	21.66 (9.18)	22.20 (14.48)	0.53 (13.91)	1.94 (-1.87 - 7.61)	1.13 (-3.23 - 5.88)	3.46 (-1.67 - 9.54)	2.83 (-2.66 - 8.14)	1.53 (-5.49 - 7.61)	1.7 (-4.64 - 7.47)
Virgin olive oil (g/day)	18.06 (18.95)	51.84 (13.22)	33.78 (21.59)	16.23 (19.00)	28.17 (22.01)	11.94 (19.55)	22.78 (24.50)	23.87 (21.46)	1.09 (20.73)	-3.41 (-13.69 - 4.26)	-6.85 (-15.95 - 2.84)	**29.14 (20.63 - 35.31)**	4.53 (-5.12 - 13.73)	**32.55 (24.06 - 41.31)**	**11.38 (2.15 - 19.56)**
Sunflower oil (g/day)	1.98 (5.25)	0.00 (0.00)	-1.98 (5.25)	0.95 (4.07)	1.03 (4.19)	0.08 (5.86)	0.99 (3.82)	2.09 (6.45)	1.10 (4.75)	1.05 (-0.88 - 2.85)	-0.19 (-1.76 - 1.67)	**-1.97 (-4.01 - (- 0.18))**	-1.22 (-3.39 - 1.25)	**-3.01 (-5.12 - (- 1.04))**	-1.04 (-3.36 - 1.31)
Fiber (g/day)	27.21 (8.71)	26.85 (7.66)	-0.36 (10.68)	26.56 (8.45)	27.44 (8.58)	0.89 (9.72)	27.10 (9.10)	26.28 (9.86)	-0.82 (10.23)	-0.76 (-3.52 - 3.74)	-1.19 (-4.34 - 3.25)	-0.33 (-3.05 - 4.19)	0.68 (-2.82 - 5.14)	0.43 (-3.80 - 4.72)	1.87 (-2.61 - 6.02)
Alcohol (g/day)	6.96 (11.43)	6.77 (11.15)	-0.19 (9.61)	8.81 (10.68)	8.28 (11.24)	-0.53 (11.49)	8.73 (15.58)	9.84 (14.31)	1.11 (11.52)	-1.44 (-7.37 - 3.84)	-0.62 (-5.61 - 5.79)	-2.68 (-8.32 - 2.18)	-2.34 (-7.07 - 3.96)	-1.24 (-5.65 - 3.04)	-1.72 (-6.63 - 3.34)

Supplementary Table S3. Student-T test comparison between baseline and 12 months gene expression (ΔCt) values (95% confidence interval).
Student-T test comparison of log_2FC values (95% confidence interval) between MedDiet-EVOO, MedDiet-Nuts and both combined against control.
Interaction term group:time (p-value) from the mixed-effects models:

- Model 1: adjusted for time, group, age, sex and education level
- Model 2: adjusted for time, group, age, sex, bmi, diabetes, dyslipidemia, physical activity, hypertension, smoking status, education level

Gene	Baseline to 12 months comparison			MedDiet vs Control comparison			Linear mixed-effects model time:group term (p-value)			
							Model 1		Model 2	
	MedDiet-EVOO	MedDiet-Nuts	Control	MedDiet-EVOO - Control	MedDiet-Nuts - Control	MedDiets combined vs Control	MedDiet-EVOO vs Control	MedDiet-Nuts vs Control	MedDiet-EVOO vs Control	MedDiet-Nuts vs Control
cd86	(-0.2019 - 0.0505)	(-0.1814 - 0.2317)	(-0.3318 - 0.0585)	(-0.169 - 0.291)	(-0.118 - 0.442)	(-0.118 - 0.33)	0,578	0,199	0,428	0,162
cdkn2a	**(-0.3591 – (-0.0118))**	(-0.0903 - 0.2726)	(-0.1866 - 0.1887)	(-0.439 - 0.066)	(-0.167 - 0.347)	(-0.287 - 0.162)	0,106	0,584	0,235	0,325
ifng	**(-0.6584 – (-0.1341))**	(-0.3522 - 0.1949)	(-0.3996 - 0.1718)	(-0.665 - 0.1)	(-0.354 - 0.425)	(-0.483 - 0.196)	0,114	0,98	0,139	0,920
il10	(-0.1633 - 0.2023)	(-0.088 - 0.389)	(-0.1864 - 0.2482)	(-0.292 - 0.269)	(-0.198 - 0.437)	(-0.212 - 0.305)	0,901	0,360	0,875	0,256
nampt	(-0.276 - 0.0999)	(-0.1961 - 0.3548)	**(-0.5094 – (-0.1026))**	(-0.055 - 0.491)	**(0.048 - 0.723)**	**(0.037 - 0.547)**	0,167	**0,018**	0,203	**0,023**
nfe2l2	(-0.1543 - 0.1044)	(-0.0503 - 0.3409)	(-0.3083 - 0.1018)	(-0.162 - 0.318)	(-0.031 - 0.528)	(-0.078 - 0.385)	0,534	0,065	0,678	0,132
nlrp3	**(0.0109 - 0.2649)**	(-0.0687 - 0.3644)	(-0.2366 - 0.1865)	(-0.081 - 0.407)	(-0.125 - 0.471)	(-0.072 - 0.407)	0,08	0,083	0,081	0,077
pik3cb	**(0.0368 - 0.2683)**	(-0.015 - 0.3666)	(-0.1943 - 0.1669)	(-0.046 - 0.378)	(-0.069 - 0.448)	(-0.03 - 0.383)	0,152	0,137	0,155	0,171
tgfb2	**(0.2268 - 0.681)**	**(0.0534 - 0.6055)**	(-0.1947 - 0.3727)	**(0.006 - 0.724)**	(-0.149 - 0.63)	(-0.02 - 0.639)	**0,035**	0,213	0,086	0,377
